# Mediating effect of social frailty on oral frailty and cognitive function in older adults: a cross-sectional study in a rural Chinese community

**DOI:** 10.3389/fnagi.2026.1826825

**Published:** 2026-09-25

**Authors:** Zhiyue Jiang, Mei Wu, Ji Tang, Lingyao Xia, Jin Hu, Quanxiang Zhou, Xing Yang, Jingyuan Yang

**Affiliations:** 1 Department of Epidemiology and Health Statistics, School of Public Health, the Key Laboratory of Environmental Pollution Monitoring and Disease Control, Ministry of Education, Guizhou Medical University, Guiyang, China; 2 Shanghai Children’s Medical Center, Guizhou Hospital, Guiyang, China; 3 Hospital of Stomatology, Shanxi Medical University, Taiyuan, China; 4 Qiannan Medical College for Nationalities, Duyun, China; 5 School of Medicine and Health Management, Guizhou Medical University, Guiyang, China

**Keywords:** cognitive function, mediating effect, older adults, oral frailty, social frailty

## Abstract

**Background:**

Cognitive function has been associated with both social and oral frailty. However, few studies have explored how social frailty and oral frailty jointly relate to cognitive function. This study aimed to explore whether social frailty plays a mediating role in the association between oral frailty and cognitive function and to examine potential gender differences in this pathway.

**Methods:**

This was a cross-sectional study design, and the data were obtained from the 2022 follow-up survey of the Health Status of Rural Older Adults (HSRO). The Mini-Mental State Examination (MMSE) was used to assess cognitive function, the Tanaka’s Oral Frailty Index-8 (OFI-8) was used to assess the risk of oral frailty, and social frailty was assessed using an operational definition that included five items: going out less frequently, rarely visiting friends, feeling unhelpful to friends or family, living alone, and not talking with someone every day. Statistical analyses were performed using hierarchical multiple linear regression analysis and Hayes’ PROCESS version 4.1 for mediation analysis in SPSS (IBM, Armonk, NY, United States).

**Results:**

Oral frailty, social frailty, and cognitive function were significantly related (*p* < 0.05). After adjusting for potential confounding variables, oral frailty and social frailty were negatively associated with cognitive function. The total effect of oral frailty on cognitive function was −0.089 (95% CI: −0.206, 0.028), and the indirect effect mediated by social frailty was −0.067 (95% CI: −0.103, −0.034), while the direct effect value was −0.022 (95% CI: −0.143, 0.098). The proportion of the indirect effect was 75%. In older men, the total effect of oral frailty on cognitive function was −0.206 (95% CI: −0.389, −0.024), and the indirect effect mediated by social frailty was −0.062 (95% CI: −0.119, −0.016). In older women, the total effect of oral frailty on cognitive function was not statistically significant, whereas the indirect effect through social frailty was −0.075 (95% CI: −0.139, −0.031).

**Conclusion:**

This cross-sectional study suggests that social frailty may mediate the association between oral frailty and cognitive function, providing statistical evidence of an association among these three factors. However, further prospective longitudinal studies are needed to validate these findings.

## 1 Introduction

With an aging population, the proportion of people aged 60 and above in the total population has increased from 16.1% in 2015 to 21.1% in 2023 in China (Nbo, 2024). Cognitive function is a vital dimension of healthy aging (Lu et al., 2021). Cognitive function declines with age, which can lead to various age-related diseases, seriously affecting older adults’ self-care ability, daily living ability, and quality of life (Raimo et al., 2024; Alhammad et al., 2026), and placing a serious burden on individuals, families, and society (Bock and Tanner, 2022). Therefore, early detection and intervention of risk factors for cognitive decline are an urgent issue at present. Studies have found that tooth loss (Mameno et al., 2024), periodontal disease (Dibello et al., 2024; Li et al., 2025), tongue pressure (Nakamura et al., 2021), and other oral health problems (Yang et al., 2024), as well as social networks (Perry et al., 2022), social support (Gui et al., 2024), air pollution (Zhu et al., 2022), and other cardiovascular factors (Rohmann et al., 2020), are closely associated with cognitive function in older adults.

Previous studies have confirmed that poor oral health is associated with cognitive decline in older adults. Tanaka et al. (2018) introduced the term “oral frailty” to describe the complex nature of poor oral health (Tanaka et al., 2018). Recent studies have found that oral frailty is associated with cognitive impairment because tooth loss reduces mastication, which may reduce blood flow to the brain, decrease neural activity in the brain, and contribute to cognitive decline (Lin et al., 2023; Chou et al., 2025; Sun et al., 2025; Panza et al., 2026; Tanaka et al., 2026). Poor oral health status can lead to periodontitis, a long-term infection caused by periodontal pathogens, and the resulting immune inflammatory responses can lead to brain damage, potentially contributing to a decline in cognitive function(Li et al., 2025; Sun et al., 2025; Farsi et al., 2026). In addition, malnutrition, a recognized risk factor for cognitive function in older adults, may be caused by decreased oral biting force, dysphagia, and other factors, thus affecting cognitive function (Nagatani et al., 2023; Xiao et al., 2023). However, there is still limited evidence of a correlation between oral frailty and cognitive function in older adults (Komatsu et al., 2021; Lin et al., 2022), and the pathways and mechanisms underlying the relationship between oral frailty and cognitive function remain unclear. It is necessary to expand the scope of the study population to provide more evidence for exploring the relationship between oral frailty and cognitive function.

Social frailty is an important phenotype of frailty in older adults(Makizako et al., 2015) and is characterized by multiple declines in social functioning, such as low social activity, a low level of social roles, and poor social relationships. Previous studies have shown that social frailty is associated with cognitive function (Zhang et al., 2025; Wang et al., 2026). It is worth noting that accumulating evidence has suggested that indicators of oral frailty, such as chewing ability, the number of existing teeth, and reduced tongue pressure, are associated with decreased social functioning (Haag et al., 2022; Iwasaki et al., 2024; Mameno et al., 2025). A Japanese study has also revealed an association between oral frailty and social frailty, and the study has shown that social frailty affects oral frailty (Hironaka et al., 2020). Longitudinal studies of community-dwelling older adults have shown that both oral frailty (Zhu et al., 2015) and social frailty (Makizako et al., 2015) negatively impact cognitive function. These studies suggest that the effects of oral frailty and social frailty on mental health are complex. Social loneliness, as an independent risk factor for dementia (Livingston et al., 2017), and poor oral health can affect the social activities of older adults, thereby accelerating the decline in cognitive function (Koyama et al., 2016). Therefore, based on the findings to date, it is hypothesized that social frailty may be a mediating variable in the relationship between oral frailty and cognitive function. In addition, a large sample survey on the prevalence of social frailty in China has shown that the prevalence of social frailty differs by gender (Qi et al., 2023). We speculate that social frailty may mediate the association between oral frailty and cognitive function, potentially being confounded or modified by gender. While previous studies have examined the direct effects of oral frailty and social frailty on cognitive functioning individually, there is limited research exploring the intricate relationships among these factors and gender-related differences.

Based on a comprehensive review of the above literature, this study proposes the following hypotheses: (1) Social frailty mediates the relationship between oral frailty and cognitive function, indicating that oral frailty indirectly affects cognitive function by promoting social frailty, and (2) the mediating role of social frailty in the association between oral frailty and cognitive function varies among older adults of different genders. This study can provide epidemiological evidence for the possible pathways and mechanisms by which oral frailty may contribute to cognitive decline.

## 2 Methods

### 2.1 Study design and participants

This is a cross-sectional study, and data were obtained from the 2022 follow-up survey of the Guizhou Rural Older Adults’ Health Study (HSRO) in China (Chen et al., 2024). The HSRO is a population-based prospective study conducted in Guizhou, China. The data were obtained using multistage cluster sampling; 12 villages were selected, the baseline survey was conducted from July to August 2019, and follow-up surveys were conducted from July to August 2022. This study analyzed the data of 1,298 individuals surveyed in 2022 (Chen et al., 2023; Tang et al., 2023; Chen et al., 2024). Eligible participants were community volunteers who were aged ≥60 years and had been long-term residents of the study area (at least >6 months). The study was approved by the Ethics Committee of Guizhou Medical University, and all participants signed the informed consent form. The flowchart is shown in Figure 1.

**Figure 1:**
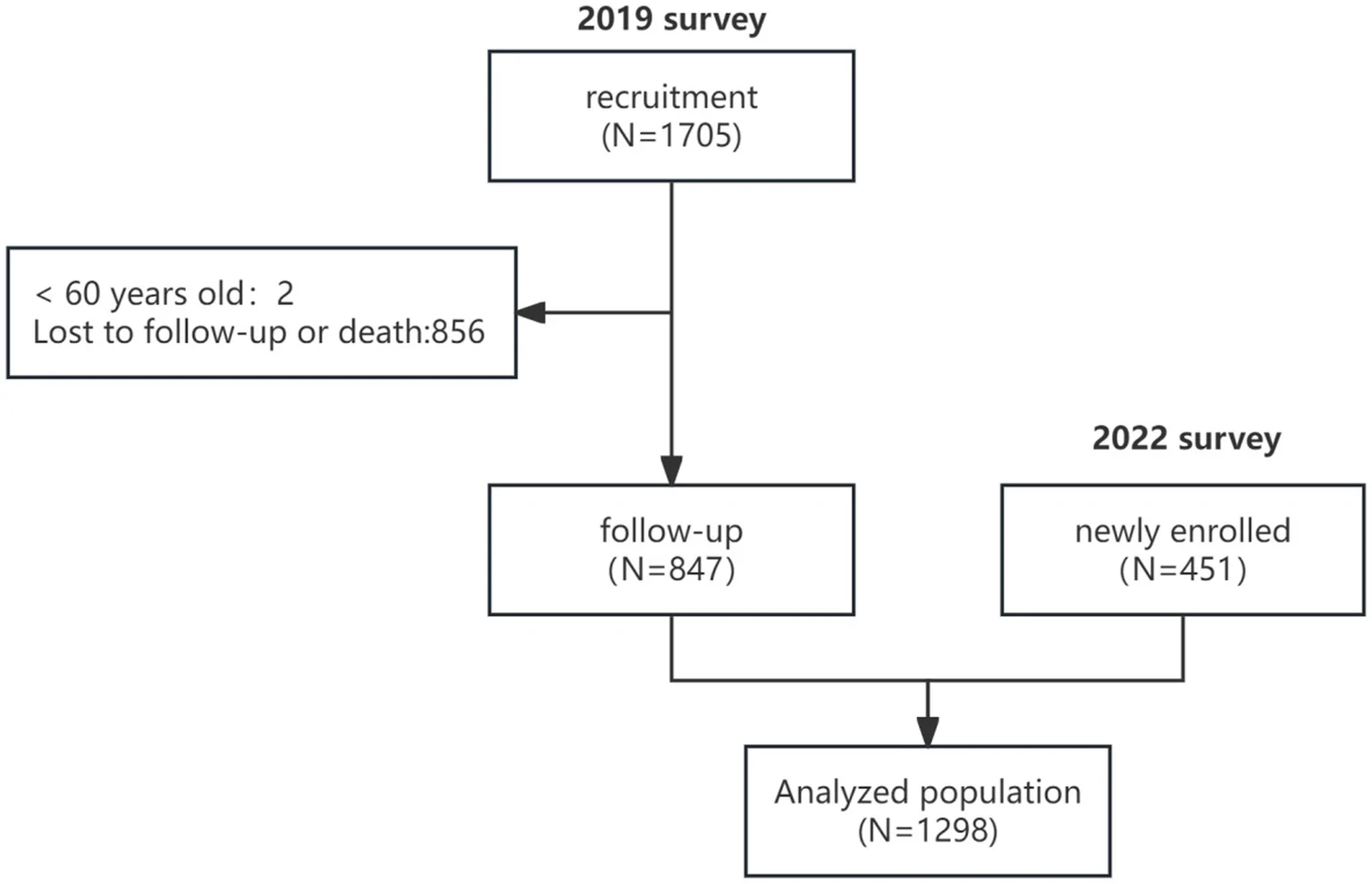
The flowchart of the participants for the analysis.

### 2.2 Cognitive function

Cognitive function was assessed by the Chinese version of the Mini-Mental State Examination (MMSE) (Folstein et al., 1975; Katzman et al., 1988). The MMSE has been widely used to assess cognitive function in older adults and has excellent reliability and validity. The test includes 11 items; the total scores range from 0 to 30 points, and a higher score indicates better cognitive function.

### 2.3 Oral frailty

Oral frailty was evaluated based on the Oral Frailty Index-8 developed by Tanaka (Tanaka et al., 2021). The eight items included “Do you have any difficulties eating tough foods compared to 6 months ago?” (yes, +2 points), “Have you choked on your tea or soup recently?” (yes, +2 points), “Do you use dentures?” (yes, +2 points), “Do you often have a dry mouth?” (yes, +1 point), “Do you go out less frequently than you did last year?” (yes, +1 point), “Can you eat hard foods like squid jerky or pickled radish?” (no, +1 point), “How many times do you brush your teeth in a day? Bushing teeth at least twice a day?” (no, +1 point), and “Do you visit a dental clinic at least annually?” (no, +1 point). The total OFI-8 score ranges from 0 to 11 points, with higher scores indicating poorer oral health. The Oral Frailty Index-8 is a useful tool for screening oral frailty (Nomura et al., 2021).

### 2.4 Operationalization of social frailty

The assessment of social frailty is based on five questions from Makizako’s report (Makizako et al., 2015). The five items are as follows: “Do you go out less frequently compared with last year?” (yes, +1 point), “Do you sometimes visit your friends?” (no, +1 point), “Do you feel you are helpful to friends or family?” (no, +1 point), “Do you live alone?” (yes, +1 point), and “Do you talk with someone every day?” (no, +1 point). The total score of social frailty ranges from 0 to 5, with higher scores indicating higher levels of social frailty.

### 2.5 Covariates

In this study, we examined several demographic characteristics of the participants through face-to-face questionnaires, including age (continuous variable in years), sex (male and female), marital status (unmarried, divorced, widowed, and married), education (continuous variable in years), occupation (farmer and non-farmer), and household income (<CNY 10,000, CNY 10,000–29,999, ≥CNY 30,000). Based on their smoking status, the participants were divided into three categories: currently smoking (defined as having smoked a total of >100 cigarettes in the past year), ever smoking (defined as having quit smoking for >6 months), and never smoking (Chen et al., 2024). Alcohol consumption was divided into three categories: currently drinking (defined as drinking on an average of ≥3–5 days per week in the past year), ever drinking (defined as having abstained from alcohol for >6 months), and never drinking. Exercise duration was divided into three categories: 0 min/day, 1–30 min/day, and >30 min/day. Body mass index (BMI) was divided into three categories: <18.5 kg/m^2^, 18.5–23.9 kg/m^2^, and ≥24 kg/m^2^(Huang et al., 2020). Activities of daily living (ADL) were assessed using the Activity of Daily Living Ability Scale (Mlinac and Feng, 2016). Participants in the study were asked about the chronic diseases they were diagnosed with.

### 2.6 Statistical analysis

In this study, SPSS version 21.0 (IBM Corp., Armonk, NY, United States) was used for data processing and descriptive statistics. Quantitative data that followed a normal distribution were described by the mean and standard deviation and analyzed using a *t*-test, while those that did not follow a normal distribution were described by the median and interquartile range and analyzed using a non-parametric test. Qualitative data were expressed as proportions and analyzed using the chi-squared test. Spearman’s correlation coefficient analysis was performed to assess the relationship between oral frailty, social frailty, and cognitive function. Furthermore, the effects of oral frailty on cognitive function were analyzed using a multiple linear regression analysis.

To analyze the relationships between oral frailty, social frailty, and cognitive function, a mediation model was tested. We hypothesized that oral frailty would predict cognitive function via social frailty. The PROCESS macro (Model 4) designed by Hayes was used to examine whether social frailty mediated the association between oral frailty and cognitive function. The mediation analysis by the PROCESS macro was based on the regression-based path analysis (Igartua and Hayes, 2021). Path analysis includes the following: (1) the path coefficient from oral frailty to social frailty (a), (2) the path coefficient from social frailty to cognitive function (b), and (3) the path coefficient from oral frailty to cognitive function in the presence of a mediator (c′) and in the absence of a mediator (c). At the same time, we adjusted for possible confounding factors, including age, sex, education, occupation, marital status, household income, smoking status, alcohol consumption status, and self-reported chronic disease. The mediation effect was quantified as a * b. Based on bias-corrected bootstrapping with 5,000 samples, we set the bootstrap confidence interval (*CI*) at 95%. Bootstrap intervals are considered statistically significant when the 95% *CI* does not contain zero. In addition, we performed a stratified analysis to examine the gender-specific mediating role of social frailty in the association between oral frailty and cognitive function.

## 3 Results

### 3.1 Characteristics of participants

Table 1 presents the characteristics of the participants. The sample consisted of 1,298 older adults, with an average age of 72.79 years (SD = 6.34). Among the participants, 536 (41.29%) were male, and 762 (58.71%) were female. Notably, 96.61% of these individuals were farmers, highlighting the predominance of this occupation within the rural Chinese community under investigation. The majority were married (63.33%), and a significant portion reported a household income between CNY 10,000 and CNY 30,000 (44.61%). Additionally, 65.25% were smokers, 61.02% consumed alcohol, 36.06% self-reported having high blood pressure, and 3.24% self-reported having diabetes. Except for age, exercise duration, high blood pressure, and diabetes, the differences in other characteristics between older men and women were statistically significant (*p* < 0.05).

**Table 1:** Characteristics of the participants (*N* = 1,298).

Variable	Overall (*N* = 1,298)	Male (*N* = 536)	Female (*N* = 762)	*t*/*χ*^2^/z	*p*-value
Age, mean (SD)	72.79 (6.34)	72.52 (6.37)	72.83 (6.32)	−0.853^a^	0.394
Education years, mean (SD)	2.79 (3.03)	4.49 (3.02)	1.59 (2.39)	18.505^a^	<0.001
Occupation, *n* (%)					
Farmer	1,254 (96.61)	501 (93.47)	753 (98.82)	27.488^b^	<0.001
Non-farmer	44 (3.39)	35 (6.53)	9 (1.18)		
Marital status, *n* (%)					
Unmarried/divorced/widowed	476 (36.67)	124 (23.13)	352 (46.19)	72.049^b^	<0.001
Married	822 (63.33)	412 (76.87)	410 (53.81)		
Household income, CNY/year, *n* (%)					
<10,000	385 (29.66)	142 (26.49)	243 (31.89)	25.427^b^	<0.001
10,000~	579 (44.61)	217 (40.49)	362 (47.51)		
≥30,000	334 (25.73)	177 (33.02)	157 (20.60)		
Smoking status, *n* (%)					
Never	343 (26.43)	317 (59.15)	26 (3.41)	733.620^b^	<0.001
Ever	108 (8.32)	98 (18.28)	10 (1.31)		
Current	847 (65.25)	121 (22.57)	726 (95.28)		
Alcohol consumption status, *n* (%)					
Never	348 (26.81)	244 (45.52)	104 (13.65)	336.431^b^	<0.001
Ever	158 (12.17)	123 (22.95)	35 (4.59)		
Current	792 (61.02)	169 (31.53)	623 (81.76)		
Exercise duration, min/day, *n* (%)					
0	479 (36.90)	187 (34.89)	292 (38.32)	4.773^b^	0.092
1–30	412 (31.74)	163 (30.41)	249 (32.68)		
>30	407 (31.36)	186 (34.70)	221 (29.00)		
BMI, kg/m^2^, *n* (%)					
<18.5	134 (10.32)	59 (11.01)	75 (9.84)	19.952^b^	<0.001
18.5–23.9	687 (52.93)	318 (59.33)	369 (48.43)		
≥24	477 (36.75)	159 (29.66)	318 (41.73)		
High blood pressure, *n* (%)					
No	830 (63.94)	356 (66.42)	474 (62.20)	2.423^b^	0.120
Yes	468 (36.06)	180 (33.58)	288 (37.80)		
Diabetes, *n* (%)					
No	1,256 (96.76)	523 (97.57)	733 (96.19)	1.915^b^	0.166
Yes	42 (3.24)	13 (2.43)	29 (3.81)		
ADL, mean (SD)	15.40 (3.26)	15.02 (3.35)	15.67 (3.19)	−3.487^a^	0.001
Oral frailty score, mean (SD)	3.41 (2.16)	3.11 (2.09)	3.62 (2.18)	−4.225^c^	<0.001
Social frailty score, mean (SD)	1.19 (1.06)	1.08 (1.00)	1.27 (1.10)	-2.871^c^	0.004
Cognitive function score (MMSE), mean (SD)	20.29 (5.96)	22.88 (5.31)	18.47 (5.72)	−13.747^c^	<0.001

^a^*t*-test, ^b^*χ*^2^ test; ^c^Mann–Whitney U-test. SD, standard deviation; CNR, Chinese Yuan; MMSE, Mini-Mental State Examination.

The oral frailty score was 3.41 (SD = 2.16), while the social frailty score was 1.19 (SD = 1.06), and the cognitive function score was 20.29 (SD = 5.96). Significant differences were observed in the scores for oral frailty, social frailty, and cognitive function between male and female participants (*p* < 0.05).

### 3.2 Correlation between the core variables

Figure 2 presents the correlations among oral frailty, social frailty, and cognitive function. In the total participant group and among male participants, oral frailty was positively associated with social frailty (*r* = 0.292, *p* < 0.001; *r* = 0.261, *p* < 0.001). Conversely, oral frailty (where a lower score indicates better oral health) was negatively associated with cognitive function (*r* = −0.133, *p* < 0.001; *r* = −0.153, *p* < 0.001), while social frailty also exhibited a negative correlation with cognitive function (*r* = −0.209, *p* < 0.001; *r* = −0.181, *p* < 0.001), with a higher MMSE score indicating better cognitive function. This result lays the foundation for the next step of multiple linear regression analysis. In female participants, oral frailty was positively associated with social frailty (*r* = 0.307, *p* = 0.001), and social frailty was negatively associated with cognitive function (*r* = −0.190, *p* < 0.001); however, no correlation was found between oral frailty and cognitive function.

**Figure 2:**
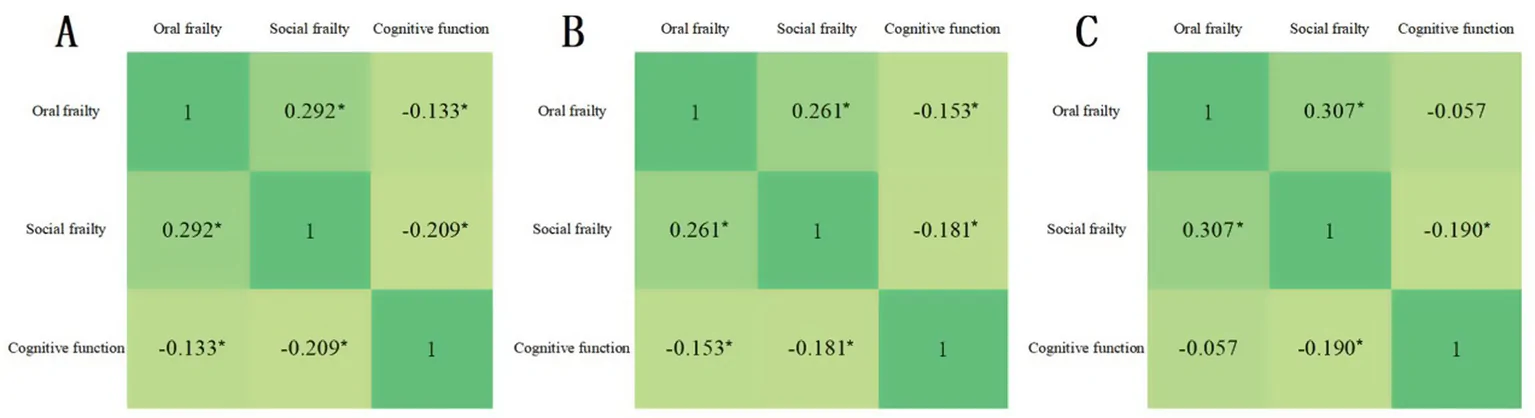
Correlation coefficients of oral frailty, social frailty, and cognitive function. Total participants **(A)**, male participants **(B)**, and female participants **(C)**. *denotes statistical significance, with *p < 0.05 (two-tailed).

We further used a multiple linear regression analysis to analyze the effects of oral frailty and social frailty on cognitive function. The effects of oral frailty on cognitive function were analyzed separately, and it was found that oral frailty had a negative effect on cognitive function (*β* = −0.349, *p* < 0.001) (Model 1), but this negative effect was weakened (*β* = −0.089, *p* = 0.136) after adjusting for relevant potential confounding factors (Model 2). After adding social frailty to Model 2, we found that oral frailty had no statistically significant effect on cognitive function (*β* = −0.022, *p* = 0.716) (Model 3). The linear regression model test showed *F* = 67.272, *p* < 0.001, and *R^2^* = 0.664. The standardized residuals met the requirements of linear regression analysis, and there was no multicollinearity among the independent variables (VIF ranged from 1.056 to 2.611). Detailed results are shown in Figure 3.

**Figure 3:**
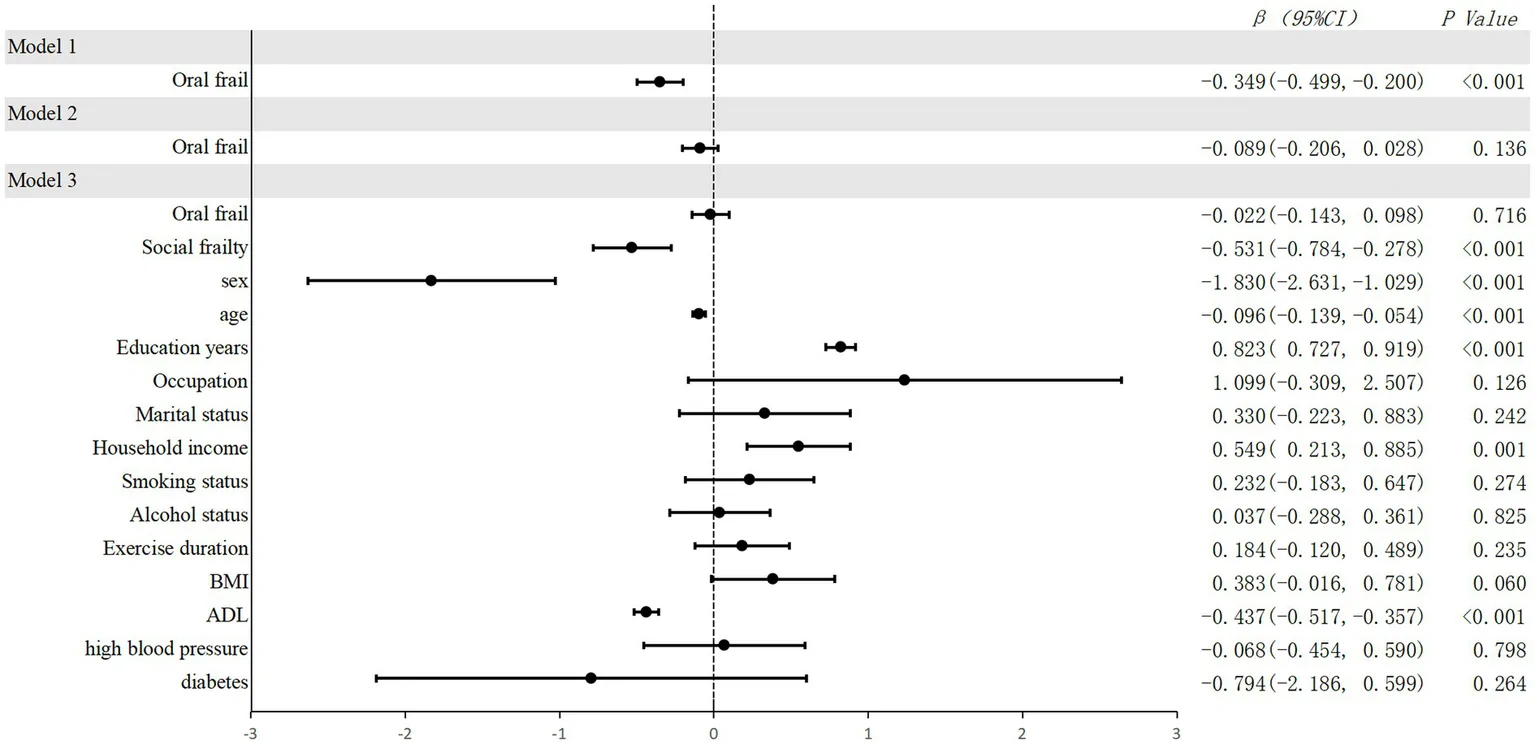
Association between oral frailty and cognitive function in older adults. Model 1 was the unadjusted model. Model 2 was adjusted for sex, age, education, occupation, marital status, household income, smoking status, alcohol consumption status, exercise duration, BMI, ADL, high blood pressure, and diabetes. Model 3 was additionally adjusted for social frailty.

### 3.3 Mediating effects of social frailty in the association between oral frailty and cognitive function

Tables 2, 3 and Figure 4 present the results of the mediating effect model analysis and the corresponding paths. In the total sample, after adjusting for sex, age, education, occupation, marital status, household income, smoking status, alcohol consumption status, exercise duration, BMI, ADL, high blood pressure, and diabetes, the total effect of oral frailty on cognitive function was −0.089 (95% CI: −0.206, 0.028). The indirect effect of social frailty was −0.067 (95% CI: −0.103, −0.034), while the direct effect was −0.022 (95% CI: −0.143, 0.098). Notably, the total effect and the direct effect of oral frailty on cognitive function were not significant, indicating that social frailty suppresses the relationship between oral frailty and cognitive function within the total population. The indirect effect accounted for 75% of the total effect.

**Table 2:** Mediation model of oral frailty, social frailty, and cognitive function.

Participant	Dependent variable	Independent variable	R	R-sq	F	*β*	t
Total	Cognitive function	Oral frailty (c)	0.658	0.433	70.008^***^	−0.089	−1.492
	Social frailty	Oral frailty (a)	0.427	0.182	20.442^***^	0.125	9.792^***^
	Cognitive function	Oral frailty (c’)	0.664	0.441	67.282^***^	−0.022	−0.363
		Social frailty (b)				−0.531	−4.116^***^
Male	Cognitive function	Oral frailty (c)	0.583	0.339	20.648^***^	−0.206	−2.222^*^
	Social frailty	Oral frailty (a)	0.394	0.155	7.364^***^	0.111	5.569^***^
	Cognitive function	Oral frailty (c’)	0.591	0.349	19.974^***^	−0.144	−1.514
		Social frailty (b)				−0.567	−2.783^**^
Female	Cognitive function	Oral frailty (c)	0.613	0.375	34.545^***^	−0.004	−0.057
	Social frailty	Oral frailty (a)	0.454	0.206	14.939^***^	0.134	7.960^***^
	Cognitive function	Oral frailty (c’)	0.620	0.385	33.328^***^	0.071	0.885
		Social frailty (b)				−0.561	−3.364^***^

Total participants: Sex, age, educational level, occupation, marital status, household income, smoking status, alcohol consumption status, exercise duration, BMI, ADL, high blood pressure, and diabetes were taken into the model as control variables. Male and female participants: Age, education, occupation, marital status, household income, smoking status, alcohol consumption status, exercise duration, BMI, ADL, high blood pressure, and diabetes were taken into the model as control variables. Standardized estimates were based on 5,000 bootstrap samples, *p < 0.05; **p < 0.01; ***p < 0.001, *β* = coefficient, 95% CI = 95% confidence interval.

**Table 3:** Mediating effect of social frailty in the association between oral frailty and cognitive function in older adults.

Participant	Effect	Estimate	SE	95% Bootstrap *CI*
Total participants	Total effect	−0.089	0.059	−0.206 ~ 0.028
	Direct effect	−0.022	0.061	−0.143 ~ 0.098
	Indirect effects	−0.067*	0.018	−0.103 ~ −0.034
Male	Total effect	−0.206*	0.093	−0.389 ~ −0.024
	Direct effect	−0.144	0.095	−0.330 ~ 0.043
	Indirect effects	−0.062*	0.027	−0.119 ~ −0.016
Female	Total effect	−0.004	0.078	−0.157 ~ 0.148
	Direct effect	0.071	0.080	−0.087 ~ 0.229
	Indirect effects	−0.075*	0.025	−0.139 ~ −0.031

SE, standard error; CI, confidence interval; LL, lower limit; UL, upper limit; *p < 0.05.

**Figure 4:**
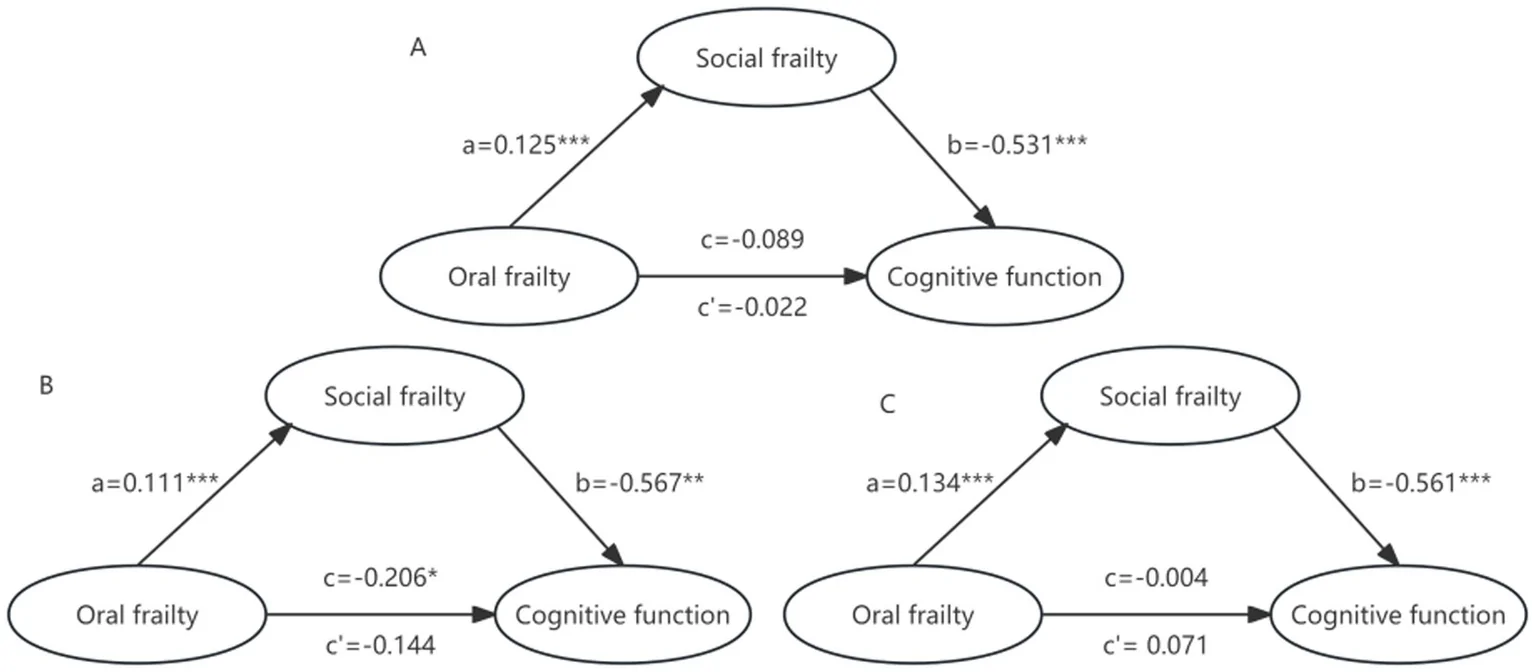
Mediating effect path of social frailty in the association between oral frailty and cognitive function. Total participants **(A)**, male participants **(B)**, female participants **(C)**. *p < 0.05; **p < 0.01;***p < 0.001.

With respect to gender differences, the full sample was divided into male (*n* = 536) and female (*n* = 762) groups for the mediating effect analyses. In the male group, social frailty completely mediated the relationship between oral frailty and cognitive function, with a mediation proportion of 30%. In contrast, among female participants, the total effect of oral frailty on cognitive function and the direct effect of social frailty were not significant; only the indirect effect was significant. The indirect effect of social frailty was −0.075 (95% CI: −0.139, −0.031), indicating that social frailty mediated the relationship between oral frailty and cognitive function.

### 3.4 Sensitivity analysis

To ensure the robustness of the research findings, we performed a sensitivity analysis. Specifically, we removed “Do you go out less frequently than you did last year?” from the assessment of oral frailty. The main research results remained consistent. This suggests that the associations observed in the initial analysis were robust (Supplementary Figure S1).

## 4 Discussion

Previous research has found that declining cognitive function is associated with oral frailty (Nagatani et al., 2023) and social frailty (Tsutsumimoto et al., 2019). This study used data from rural Chinese older adults to explore the complex associations between these factors, as well as gender differences. These findings revealed that oral frailty was not directly associated with cognitive function and that social frailty may play a mediating role in the association between oral frailty and cognitive function in older adults. Gender-stratified analyses confirmed the mediating role of social frailty, revealing a complete mediation effect among male participants. Consequently, our findings support the hypotheses we initially proposed. To the best of our knowledge, this study is one of the first investigations into the relationship between oral frailty, social frailty, and cognitive function among older adults in rural China. These findings provide valuable insights into the interplay between oral frailty and cognitive function in this demographic.

### 4.1 The association between oral frailty, social frailty, and cognitive function

This study found an association between oral frailty and cognitive function; this direct relationship diminished when the effects of social frailty were considered. These results indicate that social frailty can influence the relationship between oral frailty and cognitive function. The mediating effect analysis further elucidated the role of social frailty in the relationship between oral frailty and cognitive function. Evidence from studies has suggested that patients with oral frailty may experience difficulties with swallowing or eating (Şentürk Durmuş et al., 2026), which can impact their social functioning and subsequently be associated with a decline in cognitive function (Nagayoshi et al., 2017; Mikami et al., 2019; Nakamura et al., 2021; Jiang et al., 2022). Our research supports this view. At the same time, the manifestations of oral frailty, such as eating disorders and tongue function degradation (Matsubara et al., 2021), require treatment or may require a long-term stay at home in severe cases, thus reducing social participation and potentially contributing to a decline in cognitive function (Noguchi et al., 2019). Hironaka et al.’s (2020) study suggested that the decline in social frailty may directly affect the decline in oral frailty, and our study differs from this finding, which also suggests that social frailty and oral frailty may influence each other, although further research is needed to confirm this possibility.

However, it is noteworthy that the remaining 25% of the association between oral frailty and cognitive function may be mediated by other factors, such as malnutrition (de Sire et al., 2022), physical frailty status (Tsutsumimoto et al., 2017; Tani et al., 2022; Nagatani et al., 2023), and psychological distress (Wang et al., 2022), which warrants further exploration in future research. Our research has found that oral frailty can affect cognitive function through social frailty, suggesting that oral frailty and social frailty interventions, such as social support, which is also a surrogate indicator of cognitive reserve (Chen et al., 2024), can help the older adults maintain cognitive function and cognitive decline caused by oral frailty. Therefore, encouraging older adults who experience oral frailty to participate in more social activities, expand their social networks, and strengthen their connections to external sources of support may help maintain their cognitive reserve and potentially delay the onset and progression of cognitive decline. Our findings provide valuable insights into the prevention of cognitive decline in older adults. However, prospective studies are still needed to further confirm the reproducibility of these findings and explore the mechanisms underlying the association between oral frailty and cognitive function.

Further analysis of the mediating role of social vulnerability in the relationship between oral frailty and cognitive function by sex revealed that social frailty fully mediated this relationship in older men. However, in older women, the indirect effect of social frailty mediating oral frailty and cognitive function is significant, while the total effect and direct effect of oral frailty on cognitive function were not significant. This result suggests that social frailty may play a mediating role in the association between oral frailty and cognitive function among older women (Wen and Ye, 2014; Li and Yang, 2021). This suppression effect can be explained as follows: when oral frailty exists, it can, to a certain extent, counteract the negative impact of deteriorating oral health on cognition by seeking more social support or participating in social activities, allowing social frailty to play a “buffering” rather than “intensifying” role in this pathway (Wen and Ye, 2014). However, the “buffering” effect of social frailty may vary among older adults of different sexes; that is, the influence may be greater among women than among men, resulting in an insignificant overall effect for older women. This also suggests that cognitive intervention strategies to prevent cognitive decline in older adults may need to vary by gender. For older men, while improving oral health, particular attention should be paid to strengthening their social participation and support networks; for older women, it is necessary to enhance the sustainability of their existing social networks and be aware of the long-term impact of accumulated oral problems on cognitive function.

Although the mechanisms underlying gender differences in the impact of social frailty on the association between oral frailty and cognitive function are unclear, our findings can be explained by existing research. Unlike previous studies (Tanaka et al., 2018; Hironaka et al., 2020; Tanaka et al., 2021), our study found that the oral frailty score of older women was higher than that of older men, indicating that the oral frailty of older women was worse than that of older men. This is most likely due to the fact that the survey’s sample population consists of older ethnic minorities living in rural areas in western China. Older women in rural areas farm more frequently than men, have much lower average years of education, and may have inferior self-care and hygiene habits, resulting in poorer oral hygiene in women than in men (Tang et al., 2023). Studies in other countries have also shown that older rural women have more oral frailty than men (Kugimiya et al., 2020). Meanwhile, due to the influence of traditional Chinese cultural practices, women are more engaged in household chores and less involved in social interactions, and they may be more prone to social frailty (Wei et al., 2019). Therefore, we should pay more attention to oral frailty and social frailty in older women in rural areas to reduce the risk of cognitive function impairment.

### 4.2 Limitations

The results of this study are innovative, but there are some constraints to consider. First, the cross-sectional study design and the presence of residual confounding factors that were not accounted for may have limited our ability to infer causal relationships or establish temporal precedence. Future research should control for a broader range of potential confounding factors, such as depression and physical activity, and consider using longitudinal data to verify the directionality of the causal path. Second, as the concept of oral frailty is relatively new, there is currently no universally recognized measurement tool, and OFI-8 overlaps with some social frailty evaluation indicators. Therefore, the Chinese version of OFI-8 needs to be improved and verified for the assessment of oral frailty. Third, because the participants of this study were from rural Guizhou, China, the conclusions may not be applicable to all older adults; more studies are needed to confirm these findings. Despite these limitations, our study provides valuable information about the relationship between social frailty, oral frailty, and cognitive function in rural older adults in China.

## 5 Conclusion

This study found that social frailty plays a potential mediating role in the association between oral frailty and cognitive function among rural older adults in western China, providing cross-sectional evidence to elucidate the relationship among these three factors, although further prospective longitudinal studies are needed for verification. The research findings provide new insights into the prevention and delay of cognitive decline and have significant public health policy implications for improving the cognitive function of older adults and promoting healthy aging.

## Data Availability

The raw data supporting the conclusions of this article will be made available by the authors, without undue reservation.
