# Supplementary material for: Mediating effect of social frailty on oral frailty and cognitive function in older adults: a cross-sectional study in a rural Chinese community
Source: Front Aging Neurosci. 2026 Sep 25;18:1826825. doi: 10.3389/fnagi.2026.1826825 (PMC13649981; doi:10.3389/fnagi.2026.1826825)
Supplement: Supplementary file 1 [file Table_1.docx]

| **TABLE S1. Mediating effect of social frailty in the association between oral frailty and cognitive function in older people** | | | | |
| --- | --- | --- | --- | --- |
| **participant** | Effect | Estimate | *SE* | 95% Bootstrap *CI* |
| Total participants | Total effect | -0.109 | 0.065 | -0.238～ 0.019 |
|  | Direct effect | -0.055 | 0.065 | -0.184～ 0.074 |
|  | Indirect effects | -0.054* | 0.014 | -0.086～-0.028 |
| Male | Total effect | -0.218* | 0.102 | -0.419～-0.018 |
|  | Direct effect | -0.175 | 0.101 | -0.374～ 0.024 |
|  | Indirect effects | -0.043* | 0.021 | -0.091～-0.010 |
| Female | Total effect | -0.018 | 0.086 | -0.187～ 0.151 |
|  | Direct effect | 0.046 | 0.086 | -0.123～ 0.215 |
|  | Indirect effects | -0.064* | 0.022 | -0.111～-0.028 |
| Removed the question "Do you go out less frequently than you did last year？" from the assessment of oral frailty; SE, standard error; CI, confidence interval; LL, lower limit; UL, upper limit; **P*<0.05. | | | | |
